# Onset and Offset of Early Dupilumab Response Using Domiciliary Monitoring in Type 2 High Unified Airway Disease

**DOI:** 10.1111/cea.14550

**Published:** 2024-08-13

**Authors:** Kirsten Stewart, Chris RuiWen Kuo, Rory Chan, Brian Lipworth

**Affiliations:** ^1^ Scottish Centre for Respiratory Research, School of Medicine University of Dundee, Ninewells Hospital Dundee UK

**Keywords:** application, asthma, chronic rhinosinusitis, digital diary, dupilumab, nasal polyps, visual analogue scale

**Summary**

- Mobile phone–based devices can assess early response in objective and subjective airway outcomes with biologics.
- There was a rapid onset improvement within 2 weeks of the first dose of dupilumab.

To the Editor,

Few studies have prospectively looked in detail at concomitant upper and lower airway outcomes in patients with Type 2 (T2) high unified airway disease (UAD); severe asthma and chronic rhinosinusitis with nasal polyposis (CRSwNP). Remote follow‐up of the clinical burden of upper and lower airway disease in response to treatment, including biologics, can be challenging. There is an unmet need for a portable mobile phone‐connected device which records both upper and lower airway outcomes. Useful measures of clinical improvement include assessment of airflow obstruction as peak expiratory flow (PEF) for asthma and peak nasal inspiratory flow (PNIF) for CRSwNP, visual analogue scale (VAS) symptom scores and inhaler use. We evaluated a novel hand‐held device (Smart peak flow, Smart Respiratory Products Ltd, Imperial College, London, UK), which connects to android or iOS mobile phones via wireless Bluetooth or headphone jack.

Thirteen participants were prospectively assessed who completed e‐diary recordings, of 21 enrolled subjects with uncontrolled T2 high UAD as part of a study where patients received open‐label dupilumab 300 mg every 2 weeks [1, 2] for 12 weeks (EudraCT 2021‐005593‐25), with the primary endpoint of mannitol challenge (reported elsewhere). Patients used maintenance and reliever therapy [3] (MART 2–8 actuations daily) extra‐fine beclometasone/formoterol 100/6 μg as their inhaled corticosteroid (ICS) along with their usual intranasal corticosteroid (INCS) and entered a 4‐week run‐in period. Rescue albuterol could be used beyond the maximum MART dose of 8 actuations/day. Electronic diary recordings were made in the morning, including inhaler use (actuations/day) and the best of three readings for PNIF and PEF. VAS symptom scores for hyposmia, nasal congestion and global asthma symptoms were made by selecting a point on a 0–10 electronic scale. The study had local research ethics committee approval 21/WS/0151 and all participants gave their informed consent. Participants e‐diaries were assessed for 2 weeks prior to, and for 2 weeks following, the first dose of dupilumab. The e‐diaries were then assessed for 4 weeks following the final dose of dupilumab. Initial repeated measures analysis of variance (ANOVA) was performed followed by pairwise Student's *t*‐test with a two‐tailed alpha error of 0.05 to compare the two‐weekly mean results from 2 weeks prior to the first dose to 2 weeks following the first dose (Weeks −2 and −1 vs. Weeks 1 and 2), baseline to end of treatment (Weeks −2 and −1 vs. Weeks 11 and 12) and the 4 weeks following the final dose of dupilumab (Weeks 11 and 12 vs. Weeks 13 and 14). e‐Diary results were assessed in relation to the minimal clinical important difference values (MCID) [4, 5, 6, 7, 8, 9].

Thirteen patients completed 12 weeks of e‐diary recordings; eight had aspirin exacerbated respiratory disease (AERD) and six were males. The mean age was 53 years, mean (SEM) ICS dose was 1292 μg (102), and INCS dose was 849 μg (131) at screening. To assess onset, baseline values for e‐diary recordings 2 weeks prior to the first dose (Weeks −2 and−1) were compared to 2 weeks following the first dose of dupilumab (Weeks 1 and 2), and found mean 95% confidence interval (95% CI) improvements in PNIF of 21 L/min (3, 40) *p* < 0.05, PEF 23 L/min (2, 44) *p* < 0.05, VAS hyposmia 1.0 (0.1, 1.9) *p* < 0.05, VAS congestion 1.4 (0.8, 2.1) *p* < 0.001, VAS global asthma symptoms 1.4 (0.8, 2.0) *p* < 0.001 and MART inhaler use 1.4 (0.8, 2.0) *p* < 0.001 actuations per day. The significant improvements evident in the 2 weeks following the first injection of dupilumab are in keeping with a relatively early onset of action. Rolling two‐daily mean values illustrate the early onset of symptom and airflow improvement (Figure 1).

**FIGURE 1:**
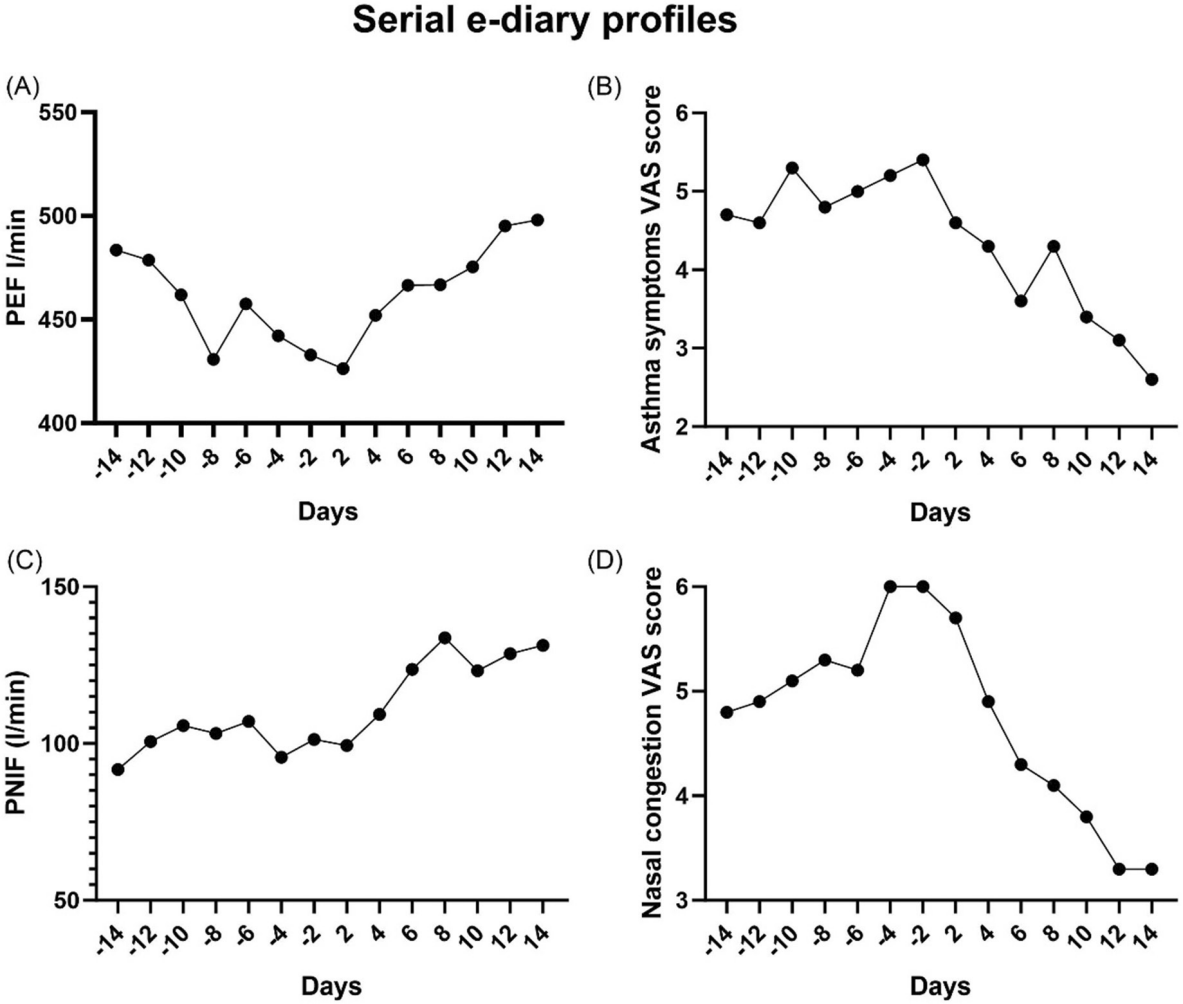
Serial e‐diary profiles for the last 2 weeks of the run‐in period (as Days −2 to −14) at baseline and the subsequent 2 weeks after the first dose of dupilumab (as Days 2 to 14). Two‐daily rolling mean values are depicted. (A) Peak expiratory flow (PEF) is noted to be increasing following first dupilumab dose, (B) asthma symptoms visual analogue scale (VAS) score (out of 10) is noted to be reducing following dupilumab, (C) peak nasal inspiratory flow (PNIF) is trending upwards following introduction of dupilumab and (D) nasal congestion VAS score (out of 10) is reducing following introduction of dupilumab. PEF, peak expiratory flow; PNIF, peak nasal inspiratory flow; VAS, visual analogue scale.

The e‐diary mean (95% CI) difference from baseline to Week 12 (Weeks −2 and −1 vs. Weeks 11 and 12) following the last dose of dupilumab showed improvements in PNIF amounting to 52 L/min (21, 82) *p* < 0.01 (>MCID of 5 L/min), PEF 53 L/min (11, 95) *p* < 0.05 (>MCID of 19 L/min), VAS hyposmia 5.3 (2.9, 7.7) *p* < 0.001, VAS congestion 4.3 (2.5, 6.2) *p* < 0.001, VAS global asthma symptoms 3.5 (1.9, 5.1) *p* < 0.001, all exceeding the VAS MCID of 2.3. Furthermore, reported MART inhaler use reduced by 2.7 actuations/day (0.3, 5.0) *p* < 0.05.

Offset e‐diary data were analysed for 4 weeks following the final dose, by comparing Weeks 11 and 12 versus Weeks 13 and 14 to ascertain whether the benefits of dupilumab waned following cessation of the drug. The mean differences in airflow obstruction and symptom scores were not statistically significant comparing responses over 2 weeks versus 4 weeks after the last dose. Rescue oral corticosteroid therapy was not required for any participant during the 12 weeks of active treatment.

The serial daily improvements noted in the early post‐treatment period indicate that there is an onset of action of dupilumab occurring within the first 2 weeks of treatment as well as no significant deterioration if a dose is missed once at steady state. We believe that using the smart peak flow e‐diary to measure serial outcomes enabled a detailed assessment of upper and lower airway obstruction, symptoms and inhaler use. This could feasibly allow virtual monitoring of asthma and CRSwNP symptoms and could reduce appointments and save patient and clinician time and hospital resources. Our preliminary data suggest the smart peak flow device may be of value in detecting early upper and lower airway responses to biologics such as dupilumab in patients with uncontrolled asthma and CRSwNP which could additionally be useful in a remote and rural setting.

## Author Contributions

B.L. and C.R.K. conceived the project and B.L., R.C. and K.S. directed it. K.S. and C.R.K. collected and analysed the data. K.S. and C.R.K. prepared the tables and figures. K.S. and B.L. drafted the manuscript. All authors contributed to revision and writing of the manuscript.

## Conflicts of Interest

Ms Stewart reports no conflicts of interest. Dr Kuo reports personal fees from AstraZeneca, personal fees from Chiesi and non‐financial support from GSK outside the submitted work. Dr Chan reports personal fees (talks) and support attending ERS from AstraZeneca, personal fees (consulting) from Vitalograph and personal fees (talks) from Thorasys. Dr Lipworth reports non‐financial support (equipment) from GSK; grants, personal fees (consulting, talks and advisory board), other support (attending ATS and ERS) from AstraZeneca; personal fees (talks and consulting) from Sanofi, personal fees (consulting, talks and advisory board) from Circassia; grants, personal fees (consulting, talks, advisory board), other support (attending ERS) from Teva; personal fees (talks and consulting), grants and other support (attending ERS and BTS) from Chiesi; personal fees (consulting) from Lupin, personal fees (consulting) from Glenmark; personal fees (consulting) from Dr Reddy; personal fees (consulting) from Sandoz; grants, personal fees (consulting, talks, advisory board), other support (attending BTS) from Boehringer Ingelheim; grants and personal fees (advisory board and talks) from Mylan outside of the submitted work; and the son of BJL is presently an employee of AstraZeneca.

## Data Availability

The data that support the findings of this study are available from the corresponding author upon reasonable request.
